# University of Tennessee

**Published:** 1888-07

**Authors:** 


### University of Tennessee—Dental Department.

The ninth annual commencement of the Dental Department of the University of Tennessee was held, in connection with the Medical Department, at the Masonic Theater, Nashville, February 22, 1888.

The degree of D.D.S. was conferred on the following graduates by Dr. Charles Dabney, President of the University:

| NAME. | RESIDENCE. | NAME. | RESIDENCE. |
|---|---|---|---|
| David H. Bell | Louisiana. | Benjamin E. Holcombe | South Carolin. |
| Freddie L. Davidson | Tennessee. | William W. Hunt | Alabama. |
| Wm. B. Elverette(honorary) | Tennessee. | William T. Mowdy | Texas. |
| Robert C. Gordon | Alabama. | James R. Pennington | Tennessee. |
| Orton P. Hart | Illincis. | | |

